# A Comparative Study upon the Therapeutic Indices of Some Natural and Synthetic Anti-inflammatory Agents

**Published:** 2011

**Authors:** Ali Khodadadi, Mohammad Hassan Pipelzadeh, Nasrin Aghel, Majid Esmaeilian, Iman Zali

**Affiliations:** 1 * Department of Immunology, School of Medicine, Ahvaz Jundishapur University of Medical Sciences, Ahvaz, Iran*; 2 *** Department of Pharmacology, School of Medicine, Toxicology Research Centre, Ahvaz Jundishapur University Medical Sciences, Ahvaz, Iran***; 3 * Department of Pharmacognosy, School of Pharmacy, Ahvaz Jundishapur University of Medical Sciences, Ahvaz, Iran*

**Keywords:** Antinflammatory agents, Cytotoxicity, Gelatinase, Matrix-metalloproteinases, Therapuetic Index, Zymography

## Abstract

**Objective(s):**

The aim of the present study was to compare the therapeutic indices of several agents used in treatment of inflammatory conditions which included: Vitamin E, hydro-alcoholic extracts of *Glycyrrhiza glabra*, *Matricaria aurea*, dexamethasone, piroxicam and diclofenac using Wehi-164 fibrosarcoma cells.

**Materials and Methods:**

Cytotoxicity evaluation was based on vital dye exclusion assay. Matrix-metalloproteinases inhibition (MMPI) was assessed by gelatinase zymography method. The collected data were used to estimate the IC_50_ (50% MMPI concentration), LC_50_ (50% cytotoxicity concentration) and the therapeutic index (LC_50_/IC_50_).

**Results:**

Among the natural anti-inflammatory agents used, *M. aurea* was the least toxic and the most effective inhibitor of MMP. Vitamin E not only increased MMP activity, but also was the most toxic of all the agents tested. Next in terms of toxicity to vitamin E was *G. glabra*. Diclofenac was more toxic than both piroxicam and dexamethasone.

**Conclusion:**

Findings from this study suggest that medicinal plants reputed to have anti-inflammatory properties are not equally effective and safe. In order to assess the implications of these findings, further *in vitro *and *in vivo* studies are needed.

## Introduction

Matrix-metalloproteinases (MMPs) are a family of multi-domain, zinc- and calcium-dependent enzymes produced by a wide range of stromal and inflammatory cells and are capable of degrading a variety of components of the extra cellular matrix. The role of MMPs has been well established in a variety of inflammatory diseases such as arthritis (1) cardiovascular (2), metastasis of cancer (3) and in angiogenesis (4). Furthermore, MMPs (mainly membrane-type MMPs) have an important physiological role in embryo implantation, bone remodeling and organogenesis (5). MMPs can be constitutive (homeostatic MMPs), which are switched on in most cells under steady-state conditions, or inducible (pro-inflammatory MMPs), which have more complex promoter region and regulated by various agonists like in invasion of cancer cells. These findings highlight the complex nature of MMPs. 

The biological activity involving MMPs are always dependent on maintaining a balance between proteinases and natural tissue inhibitors of matrix metalloproteinases (TIMPs), such as serum _2_-macroglobulin (6), monoclonal antibodies, and small molecules (both synthetic and natural products). Modulation of MMP activity, therefore, seems to be a useful therapeutic option in the treatment of both chronic and acute inflammatory conditions in which excess MMP activity has been implicated. However, despite their high potencies, MMPIs act non-specifically which leads to side effects (7). The search for finding more selective and potent agents for clinical application in various inflammatory conditions is still continuing.

On the other hand, different classes of anti-inflammatory drugs have been employed for treatment of a variety of inflammatory diseases which includes steroidal and non-steroidal as well as drugs obtained from medicinal plants (8). Because of their efficacy in the treatment of various inflammatory conditions, both steroidal and non-steroidal anti-inflammatory drugs (NSAIDs) are of the most frequently used classes of medicines in the world and account for more than 5% of all prescribed medications (9). In addition, the use of these agents is associated with various side effects such as gastrointestinal complications, and with prolonged use of corticosteroids, reduction in immune responses and dysfunction of HPA axis. Furthermore, there are few and limited controlled *in vitro* or clinical trials on the effects of herbal products. Therefore, their safety and efficacy have been always questioned by the approving authorities. However, anecdotal evidences suggest that herbs can be useful in the treatment of a variety of inflammatory conditions such as periodontal diseases (10) and in experimentally-induced granulation pouch (11). Among the ancient medicinal herbs that are still used for various inflammatory conditions are *Glycyrrhiza glabra* (12) and *Matricaria aurea*. While *G. glabra* has been suggested to be useful in the treatment of cancer (12), and in protection of liver following experimentally induced carbon-tetrachloride toxicity (13). *M. aurea* has been shown to have strong antioxidant properties (14) and traditionally used in case of urinary retention, urinary tract infections and kidney and testicle pain (15). Vitamin E, another potentially useful antioxidant agent, was found to contribute to the prevention of atherogenesis and restenosis arising from vascular injury associated with MMP-1 activation via its antioxidant activity (16).

Despite these findings, no previous study has attempted to correlate the MMPI activities of these synthetic and natural anti-inflammatory agents with their cytotoxicity and estimation of their therapeutic indices. The aims of the present study were twofold: Firstly, to compare IC_50_ values of dexamethasone, piroxicam and diclofenac as well as three natural agents reported to have anti-inflammatory properties: vitamin E, hydro-alcoholic extract of *G. glabra *and *M. aurea*. Previous studies have shown a close relationship between MMP_2_ and MMP_9_ inhibition with anti-inflammatory properties of some of these agents used in this study (17). Secondly, the cytotoxicity produced by the above agents was compared by estimation of their LC_50_ values. The aim of such comparative studies was to provide scientifically-based evidence of the therapeutic safety margins of agents which may be administered in the treatment of an inflammatory disease. Prior to becoming a drug for therapeutic use, a medicinal agent, synthetic or of herbal origin, vigorous *in vitro* and *in vivo* studies are needed to determine its safety and efficacy.

## Materials and Method

***Cell culture***

The fibrosarcoma (Wehi-164, National Cell Bank of Iran, Pasteur Institute, ) cell line was seeded at initial density of 20,000 cells/well in 96-well tissue culture plate. Cells were maintained in RPMI-1640 medium supplemented with 5% fetal calf serum, penicillin (100 units/ml) and streptomycin (100 µg/ml) under 5% CO_2_, 37 °C and saturated humidity (18).

Preparation of hydro-alcoholic extract of *G. glabra and M. aurea *(purchased from commercial market). The plant materials were identified by an expert botanist. The roots of *G. glabra* were cut and grinded into powder. Oven-dried *M. aurea* flowers were directly grinded. For each stage of extraction, 100 g of the powdered plant product was moistened for 2 hr with 70% ethanol solution and allowed to swell before being placed in one of a series of percolation chambers. The materials were repeatedly rinsed with 70% alcoholic solvent until the solvent became clear which required 24 to 48 hr of percolation. The collected percolation products were vacuum-dried and the extracts were dissolved and diluted in PBS to the desired concentrations. Percentages obtained for both extracts were approximately 7% (19). 

***Dose–response analysis***

Sterile solutions of triplicate of two fold dilutions in PBS were prepared from pure substances of disodium dexamethasone sulphate (purchased from Daru Pahksh Pharmaceutical Co. Iran), piroxicam (purchased from Poorsina Pharmaceutical Co. Iran), diclofenac Na (purchased from Daru Pahksh Pharmaceutical Co, Iran), at concentrations of 10–200 µg/ml, vitamin E (*dl-*-tocophirol acetate) (1 to 50 µg/ml) (purchased from Osveh Pharmaceutical Co, Iran), *and G. glabra* and *M. aurea* extracts (8 to 8000 µg/ml), were transferred to cultured cells. Non-treated cells, exposed to PBS alone, were used as control. Cells were cultured for 24 hr and then were subjected to colorimetric assay.

***Vital dye exclusion cytotoxicity assay***

Cell cultures were washed three times with ice-cold PBS, followed by fixation in a 5% formaldehyde solution. Fixed cells were washed three times and stained with 1% crystal-violet. Stained cells were washed, lysed and solubilized with 33.3% acetic acid solution. The density of developed purple color was read at 580 nm by colorimetric method (Tecan, , Remote Touch Screen, Austria). The extent of color density was considered as the degree of cytotoxicity relative to background reading values for the control untreated cell culture (0% cytotoxicity). Furthermore, reference background readings for the plant extracts were subtracted from the relevant plant readings. Data from linear regression analysis of concentration-response curves were used in order to estimate the concentration that produced 50% cytotoxicity (LC_50_).

***Zymography***

This technique was used to determine gelatinase A (MMP-2) and gelatinase B (MMP-9) activity in conditioned media according to modified Heussen and Dowdle method (18). Briefly, aliquots of conditioned media were subjected to electrophoresis in (2 mg/ml) gelatin-containing polyacrylamide gels in the presence of sodium dodecyl sulfate polyacrylamide gel electrophoresis (SDS–PAGE) under non-reducing conditions. The gels underwent electrophoresis for 3 hr at a constant voltage of 80 V. After electrophoresis, gels were washed and gently shaken in three consecutive washings in 2.5% Triton X-100 solution to remove SDS. Gel slabs were then incubated at 37 °C for 24 hr in 0.1 M Tris–HCl gelatinase-activation buffer (pH= 7.4) containing 10 mM CaCl_2 _and subsequently stained with 0.5% Coomassie Blue. After intensive destaining, proteolysis areas appeared as clear bands against a blue background. Quantitative evaluation of both surface and intensity of lysis bands were relatively compared to non-treated control wells and expressed as % MMPI relative to control gelatinase activity. Values obtained from standard references for gelatinase A and B and IC_50_ values were determined from the linear regression analysis of concentration-response curves. 

***Statistical analysis***

All experiments were performed in triplicates. The graphic data were presented as percentage of mathematical means relative to control baseline values before addition of agents tested ± SEM. In evaluation of differences between recorded data the *P*-value< 0.05 was considered significant using ANOVA followed by Dunnets' *post-hoc* test. Prism software was employed to produce a linear regression analysis from the concentration-response curves and determination of both LC_50_ and IC_50_ by extrapulation of the mathematical module: *Y=bX + a, *where the coefficients a (intercept) and b (slope) and X is the corresponding concentration at which Y (LC_50_ or IC_50_) occurred. 

## Results

***Comparison of cytotoxic effects (LC50 values)***

**Figure 1.**
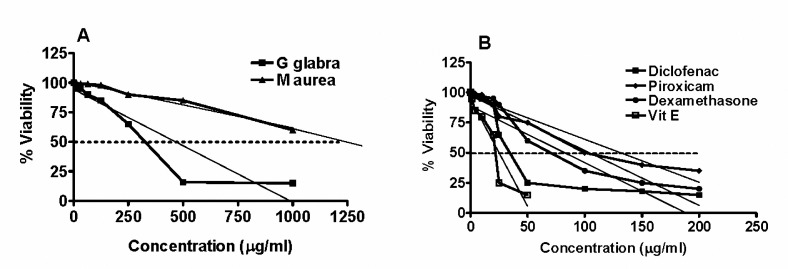
Mean percentage±SEM of viable Wehi-164 fibrosarcoma cells, relative to their viability in untreated control cells, following 24 hr incubation with varying concentrations of A- hydro-alcoholic extract of * Glycyrrhiza glabra* and *Matricaria* *aurea*, B- dexamethasone, piroxicam, diclofenac, and vitamin E. The 50% cytotoxicity (LC_50_) value for each agent was estimated by linear extrapolation method using Prizm software.

Figure 1and Table 1show the concentration-percentage relationships of viable Wehi-164 cells curves relative to untreated control group and the corresponding LC_50_ values of diclofenac, piroxicam, and dexamethasone, respectively. LC_50_ values for vitamin E, diclofenac, dexamethasone and piroxicam were 25, 82.3, 104, 131 µg/ml, respectively. Corresponding LC_50_ values for *G. glabra *and *M. aurea* hydro-alcoholic extracts were 465 and 1305 µg/ml, respectively (Table 1 and Figure 1). LC_50_ values of the plant extracts against Wehi-164 fibrosarcoma, as a sensitive cell line (17), were significantly lower relative to vitamin E, dexamethasone, piroxicam, and diclofenac (Table 1). 

***Comparison of inhibition of MMP (MMPI) activities***

**Figure 2.**
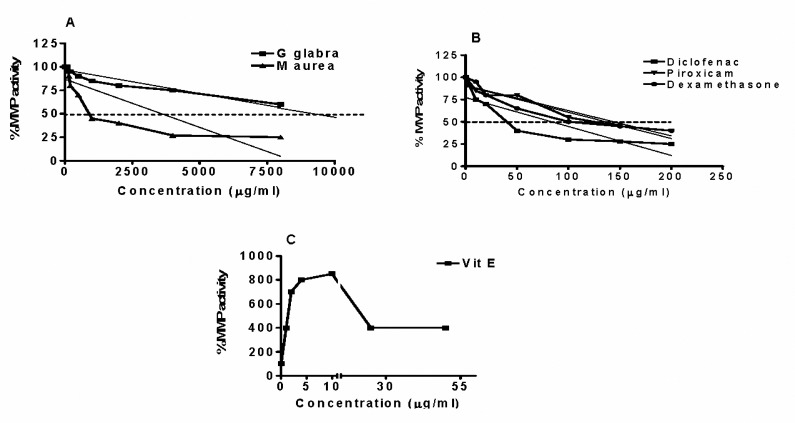
Mean percentage±SEM changes in metalloproteinase activity relative to basic untreated control Wehi-164 fibrosarcoma cells following 24 hr incubation with varying concentrations of A- hydro-alcoholic extract of * Glycyrrhiza glabra* and *Matricaria* *aurea*; B*-* dexamethasone, piroxicam and diclofenac; and C- vitamin E. The 50% MMPI (IC_50_) values were extrapolated by linear extrapolation method using Prizm software.

The IC_50_ value of hydro-alcoholic extract of* G. glabra* was 2.59 fold higher than that of *M. aurea* (9288 versus 3588 µg/ml) (Figure 2 and Table 1), while the corresponding values for diclofenac, dexamethasone and piroxicam were 83.6, 136.3, 143.8 µg/ml respectively (Table 1). The percentage of MMPI activity of diclofenac, over all concentration range used (10–200 µg/ml) was significantly greater than those recorded for piroxicam and dexamethasone (Figure 1). Vitamin E produced an increase in the MMP activities at the concentration range of 1 to 10 µg/ml. This increase in MMP activity was found to be maximal at 10 µg/ml reaching 8 fold higher than baseline value recorded for untreated control cells. At concentrations above 10 µg/ml, vitamin E produced a gradual reduction from the maximum reaching 400% of the baseline value at 50 µg/ml (Figure 1).

***Comparison of the therapeutic indices (LC50 / IC50 ratios)***

The ratios of the cytotoxicity (LC_50_) to anti-inflammatory effectiveness (IC_50_) of the studied agents were used as measure for the therapeutic index. The results showed that vitamin E was the most toxic of all agents used; not only it increased the MMP activity but also it had the lowest LC_50_ value at 25 µg/ml. On the other hand, *G. glabra* was found to be the second in line to vitamin E in terms of its LC_50_/IC_50 _ratio, having a therapeutic index of 0.05, while diclofenac was the safest with a therapeutic index of 0.98 (Table 1). 

**Table 1.** Mean linear extrapolation values of 50% cytotoxicity values (LC_50_ values, µg/ml) and 50% metalloproteinase inhibitory (IC_50_, µg/ml), and their calculated therapeutic indices (LC_50_/IC_50_) of hydro-alcoholic extract of *Glycyrrhiza glabra* and *Matricaria* *aurea*, dexamethasone, piroxicam and diclofenac on Wehi-164 fibrosarcoma cells.

Test/ Agent	*G. glabra*	*M. aurea*	Dexamethasone	Piroxicam	Diclofenac
LC _50_	465	1450	104	131	82.3
IC _50_	9288	3584	136.3	143.8	83.6
LC_50_/ IC_50_ Ratio	0.05***	0.40^+++^	0.76^+^	0.91	0.98

***P< 0.001 of *G. glabra* relative to other treatment groups. ^+++^*P*< 0.001 of *M. aurea* relative to other groups. +*P*< 0.5 between dexamethasone and piroxicam and diclofenac. For statistical evaluation, ANOVA followed by Dunnets' post hoc tests were used. LC_50_/IC_50_ ratios were calculated by dividing the LC_50_ with IC_50_ values which were obtained by interpolation from linear regression analysis of the dose response cures obtained from zymography and colorimetric methods, respectively.

## Discussion

In this study, using Wahi-164 fibrosarcoma cell line, zymography method of analysis and vital dye cytotoxicity exclusion assay, the therapeutic indices (LC_50_/IC_50_ ratios) of two NSAIDs namely diclofenac, piroxicam, and a steroidal agent, dexamethasone, were measured and compared with those produced by vitamin E and hydro-alcoholic extracts of *G. glabra *and *M. aurea*. The results showed that the descending order of IC_50_ values (MMPI efficacy) was in the following sequence: diclofenac > dexamethasone > piroxicam > *M. aurea > G. glabra. *Contrary to these agents, vitamin E (10 µg/ml) produced an eight fold increase in MMP activity. Furthermore, the descending order of LC_50_ values (cytotoxic effect) was: vitamin E > diclofenac > dexamethasone > piroxicam >* G. glabra> M. aurea*. However, when comparative therapeutic indices of these agents were calculated, diclofenac was found to be the most efficacious and the least toxic among the tested compounds. The descending order of these ratios was: diclofenac > piroxicam > dexamethasone > *M. aurea *>*G. glabra* > vitamin E. 

Pharmacologically NSAIDs are clinically proved effective anti-inflammatory drugs and are thought to act through inhibition of cyclooxygenase enzyme. Other studies have linked their anti-inflammatory action with their MMPI effects (17). However, other mechanisms explaining their cytotoxicity have been proposed: While Saunders *et al* (21), postulated that NSAIDs cause depletion of intracellular polyamines - naturally occurring growth factors found in all cells - thus leading to decrease in growth and death in cancer cells while others suggested a direct increase in permeabilization of cell membrane to calcium ions (22). 

Findings from our study showed a relative close correlation between MMPI activities and the cytotoxic effects of the tested NSAIDs. However, is there any direct relationship between cytotoxicity and MMPI activity? This seems to be unlikely for three reasons: Firstly, MMPs are known to be secretory products released under both physiological and pathological conditions, while cellular death is orchestrated by intracellular processes leading to apoptosis or necrosis. Secondly, following the disappointing outcomes of MMPIs as modulators of cellular growth in treatment of cancer (23), these agents have been advocated, with different degrees of success, in the treatment of various acute and chronic inflammatory conditions (24). Thirdly, as shown from our results, vitamin E was found to cause an increase in MMP activity, yet it also produced cytotoxicity. These observations suggest that inhibition of MMP activity is not related to the cytotoxicity and these two processes are orchestrated independently by different mechanisms. 

The results from estimated LC_50_ and IC_50_ values indicate that there are differences between the two hydro-alcoholic extracts from *G. glabra *roots and *M. aurea* flowers. *G. glabra* was three fold more cytotoxic (LC_50_ 465 versus 1450) and 2.6 fold less potent as an anti-inflammatory natural product (IC_50_ of 9288 versus 3584) than *M. aurea*. Consequently, *M. aurea* is shown to be a safer and a more effective anti-inflammatory agent of natural origin than *G. glabra* but less effective than conventionally used NSAIDs and dexamethasone. Prudently, further *in vivo *and *in vitro *studies on the anti-inflammatory properties of the active constitutes of this these medicinal plant are needed. On the other hand, the findings from examining extract from* G. glabara *comes in agreement with a recent report in which the aqueous extract of *G. glabra* was found to have significant,* in vivo* and *in vitro *anti-proliferative and anti-angiogenesis effects on Ehrlich ascites tumor cells (12). Based on these findings it seems that the extract from this medicinal plant has a potential role as a supplemental source in cancer therapy.

Despite extensive researches on vitamin E, its exact role in prevention or treatment of a given inflammatory disease and on the mechanism by which it produces the proposed effects is not clearly defined. While it has been regarded merely as an antioxidant (25), others dispute this as the main mechanism of action and argue that it has no *in vivo* protective effect against oxidative damage or prevention of diseases which have at their basis an oxidative insult (26). Furthermore, in our study, in contrast to other tested agents, findings from vitamin E experiments showed that it increased MMP activity. This finding comes in agreement with those reported by Chakraborti *et al* where pretreatment of the microsomes with vitamin E (1 mM) preserved the increase in MMP-2 activity, Ca^2 +^ATPase activity and also ATP-dependent Ca^2 +^ uptake in the microsomes following exposure to peroxynitrite (27). 

The other unexpected aspect of vitamin E was its cytotoxic property. It was found to have the lowest LC_50_ and accordingly was the most toxic of all the tested agents. Anti-proliferative effects of vitamin E were previously reported for neuroblastoma cells (28) and different formulations of vitamin E produced varying degrees of apoptosis (29,30). The exact mechanism that mediate vitamin E cytotoxic effects can not be clarified from present experiments and further works need to be undertaken. However, it is possible that vitamin E may have an indirect effect via interaction with the pro-antioxidants in the culture media. These effects were not tested in our experiments and need further assessments. 

MTT, LDH leakage, and dye exclusion assays are the most commonly used cytotoxicity assays in *in vitro* toxicological studies on different cell lines. However, previous studies have shown that these tests produced variable results, which are attributed to the differences in the sensitivities of the cell lines employed as well as to the agents used (31α_2 _and MMP_9_ isoenzymes produced by Wehi 164 fibrosarcoma cells which are known for their high yield of MMP secretory activity (32).

It is well known that the duration of incubation, the concentrations of the test agents as well as the type of employed dye are among the important detrimental experimental conditions that need to be considered carefully when performing cytotoxicity tests (31,33). In order to limit the effects of these confounding factors, we allocated the 24 hr as the fixed time point and any cellular damage that has occurred at the IC_50_ or EC_50 _concentrations of the tested agents was assumed to be similar. Previous studies using 24 hr incubation period was found reliable for measurement of IC_50_ values (33) and, in our preliminary experiments, this time point was found to be suitable and gave reproducible results. These findings are merely a small step in proposing a simple method for evaluating the safety and efficacy of various anti-inflammatory agents both from synthetic and natural sources.

What is the relevance of these findings to clinical practice? Inhibition of a selected MMP may have a potential beneficial role in the treatment of inflammatory-based conditions. To achieve such a target, ideally we need to have a drug which selectively, safely and effectively inhibits a specifically implicated MMP. In this *in vitro *study we used the therapeutic index as an index for assessment of safety profile and showed that, among the tested agents, the hydro-alcoholic extract of *M. aurea *was a safer inhibitor of MMP-2 and MMP-9 than vitamin E* and G. glabra*. Prudently, more detailed studies on the active constituent(s) of this medicinal plant need to be undertaken in order to utilize it in clinical practice. On the other hand, herbal treatments are becoming increasingly popular, are often used in complementary medicine for treatment of various ailments and conditions, and are often associated with an intuitive feeling that "naturalness" is a guarantee of harmlessness. The findings from this study have shown that a product from natural origin does not simply means that it is a safe agent. Using these simple *in vitro* tests, we have shown that vitamin E and *G. glabara* are potent cytotoxic agents and highlight the importance of vigorous preclinical investigations on the possible adverse effects associated with herbal or natural agents. Such studies may pave the way for better understanding of the potential role of the herbal agents as therapeutic tools in modern health systems.
